# Magneto-Sensitive Adsorbents Modified by Functional Nitrogen-Containing Groups

**DOI:** 10.1186/s11671-016-1273-4

**Published:** 2016-02-03

**Authors:** Inna V. Melnyk, Karolina Gdula, Andrzej Dąbrowski, Yuriy L. Zub

**Affiliations:** Department of Surface Chemistry of Hybrid Materials, Chuiko Institute of Surface Chemistry, National Academy of Sciences of Ukraine, 17, General Naumov str., Kyiv, 03164 Ukraine; Faculty of Chemistry, Maria Curie-Sklodowska University, 3, Maria Curie-Sklodowska Sq., Lublin, 20-031 Poland

**Keywords:** Magnetite nanoparticles, Polysiloxane shells, Nitrogen-containing functional groups, Copper(II) ions adsorption

## Abstract

In order to obtain amino-functionalized silica materials with magnetic core, one-step synthesis was carried out. Several materials, differ in number and structure of amino groups, were synthesized on the basis of sol-gel method. The synthesized materials were examined by several analytical techniques. The presence and content of amino groups were measured by using Diffuse Reflectance Infrared Fourier Transform (DRIFT) spectroscopy and acid-base titration, respectively. Specific surface areas were measured by nitrogen/adsorption desorption isotherms. It was proved that sol-gel approach leads to obtain materials with high content of amino groups built into their surfaces (in the range 1.6–2.7 mmol/g). As-obtained materials were tested as potential adsorbents for copper(II) ions. The received maximum adsorption capacities were in the range 0.4–0.7 mmol/g.

## Background

A number of industrial enterprises have increased significantly with the simultaneously development of society. Consequently, a lot of hazardous compounds, such as heavy metal ions or pharmaceuticals, enter into the natural environment. Thus, there is a need to remove those substances from natural environment. Therefore, modern methods which are characterized by high efficiency, environmentally safe and are relatively inexpensive, should be proposed to remove such substances, especially from water and wastewaters. To separate heavy metal ions from aqueous solutions, a lot of methods based on using nanocomposites of magnetite are used [1, 2]. Many papers are devoted to the removal of copper ions [3–5], lead [4–6], cadmium [4, 5], and so on. From the one hand, using the magnetic nanoparticles coated with functionalized silica materials allows to adsorb onto their surface many organic or inorganic compounds; on the other hand, separation of adsorbent with the adsorbed compounds from solution can be easily carried out by using permanent magnet.

In the literature, many variety of methods, describing coating Fe_3_O_4_ nanoparticles, can be found, e.g., direct modification of magnetite by amino-containing silanes [5, 7] and magnetite coated with polysiloxane layer and their further modification by amino containing silane [3, 4, 8, 9] as well as one-step technique using tetraethoxysilane and N-containing silanes [10, 11]. The first method does not allow to obtain tight and continuous layer covered magnetite nanoparticles. What is more, such approach does not result in the introduction of large amounts of amino groups into silica structure, and in consequence, leads to obtain very small adsorption properties of final materials. While the second method sometimes includes many steps and requires using very expensive reagents, the third method is a one-step technique and if it is necessary, allows introducing more than one functional group, differing in their chemical nature (Table 1). In this work, we proposed one-step synthesis for obtaining functionalized magnetic silica materials. Such materials were tested as effective adsorbents of copper(II) ions from aqueous solutions.

**Table 1:** Composition of initial reactants and properties of magneto-sensitive adsorbents

Sample	Functional groups	Molar ratio of components Fe_3_O_4_/TEOS/N-silane/R-silane	*C*_f.gr._, mmol/g	*S*_sp._, m^2^/g
A	≡Si(CH_2_)_3_NH_2_	1/15.5/2.7	2.2	122
AM	≡Si(CH_2_)_3_NH_2_/≡SiCH_3_	1/15.5/1.3/1.3	1.6	29
AP	≡Si(CH_2_)_3_NH_2_/≡Si(CH_2_)_2_CH_3_	1/15.5/1.3/1.3	1.6	43
DA	≡Si(CH_2_)_3_NH(CH_2_)_2_NH_2_	1/15.5/2.7	2.7	160
BA	[≡Si(CH_2_)_3_]_2_NH	1/15.5/2.7	1.9	33

*C*_f.gr._—concentration of amino groups present in adsorbent structure; *S*_sp._—specific surface area; N-silane—functional monomer having amino groups; R-silane—functional monomer having aliphatic chains

## Methods

### Chemicals and Reagents

Iron(II) chloride, FeCl_2_∙4H_2_O (Sigma-Aldrich, 99 %); iron(III) chloride, FeCl_3_∙6H_2_O (Sigma-Aldrich, 98 %); ammonium hydroxide, NH_4_OH (Aldrich, 25 % aqueous solution); tetraethoxysilane, Si(OC_2_H_5_)_4_ (TEOS, Aldrich, 98 %); 3-aminopropyltriethoxysilane, (C_2_H_5_O)_3_Si(CH_2_)_3_NH_2_ (APTES, Aldrich, 99 %); methyltriethoxysilane, (C_2_H_5_O)_3_SiCH_3_ (MTЕS, Aldrich, 99 %); *n*-propyltriethoxysilane, (C_2_H_5_O)_3_Si(CH_2_)_2_CH_3_ (PTES, Fluka, 97 %); *N*-[3-trimethoxysilylpropуl]ethylendiamine, (CH_3_O)_3_Si(CH_2_)_3_NH(CH_2_)_2_NH_2_ (TMPED, Aldrich, 97 %); bis[3-(trimethoxysilylpropyl)]amine [(CH_3_O)_3_Si(CH_2_)_3_]_2_NH (BTMPA, Aldrich, 90 %); ethanol, C_2_H_5_OH (EtOH, 96 %); and ammonium fluoride, NH_4_F (analytical grade, Reahim, Ukraine) were used as received, without further purification.

Copper(II) nitrate, Cu(NO_3_)_2_∙3Н_2_О (Merck, 99.5 %). Ammonium chloride, NH_4_Cl; sodium nitrate, NaNO_3_; sodium chloride, NaCl (chemically pure, Macrochem, Ukraine). Nitric acid, HNO_3_; hydrochloric acid, HCl; sodium hydroxide, NaOH; ethylenediaminetetraacetic acid EDTA, C_10_H_16_N_2_O_8_ (fixanal concentrates, Cherkasy State Chemical Plant, Ukraine). Methyl orange, C_14_H_14_N_3_NaO_3_S (analytical grade, Reahim, Ukraine); and murexide, C_8_H_8_N_6_O_6_ (analytical grade, Reahim, Ukraine) were used.

### Syntheses

Each of examined amino-functionalized silica adsorbents with magnetic core was synthesized on the basis of sol-gel procedure, which is detailed described in papers [10, 11]. The magnetite nanoparticles were obtained by co-precipitation of iron(II) and (III) salts in a basic medium, reported by [12]. Briefly, iron chlorides(II) and (III) (in molar ratio: Fe^2+^/Fe^3+^ = 1/2) were dissolved in 450 mL of distilled water at 80 °C, under nitrogen flow. Next, 50 mL of ammonia solution was slowly added to the mixture. The black magnetite precipitate was produced in a few seconds and kept under 80 °C and mechanical stirring, during 30 min. After this time, the heating was switched off, and in 10 min more, the stirrer was also switched off. After cooled to the room temperature, the magnetic nanoparticles were separated from solution by decantation on permanent neodymium magnet. In order to remove unreacted reactants, the magnetite particles were cleaned by repeated cycles of water, to obtain final pH = 6.

In order to prepare silica layers onto as-prepared magnetite nanoparticles, sol-gel procedure was employed. This method is based on hydrolysis and condensation of TEOS and a proper functional monomer (having amino groups), in the presence of catalyst. In order to prepare various materials differing in both, type and amount of amino groups built in the adsorbent structure, and presence of additional aliphatic chain, five different materials were synthesized (three single- and two bifunctional). The resulting samples were labeled as follows: Fe_3_O_4_/TEOS/APTES—A, Fe_3_O_4_/TEOS/APTES/MTES—AM, Fe_3_O_4_/TEOS/APTES/PTES—AP, Fe_3_O_4_/TEOS/TMPED—DA, and Fe_3_O_4_/TEOS/BTMPA—BA. Briefly, 0.75 g of magnetite nanoparticles was placed into the three-neck flask and dispersed in 62.5 mL of distilled water. To ensure better particle dispersion, ultrasound treatment was used. In a separate beaker, calculated amount of a proper functional monomer was mixed with ethanol (3 mL) and ammonium fluoride (1.9 mL of 1 % aqueous solution), acting as a catalyst role. After 5 min, the monomer solution was added to magnetite nanoparticles and stirred during 5 min. Next, 11.3 mL of TEOS was added dropwise. The stirring was continued during 6 h. After this time, the reaction was completed. In order to remove unreacted compounds and impurities, the amino-functionalized silicas with magnetic core were cleaned by repeated cycles of decantation on the magnet and redispersion in distilled water, (3 × 50 mL) and ethanol (2 × 50 mL). As-prepared adsorbents were dried overnight in the oven at 100 °C. In the case of bifunctional samples (AM and AP), mixtures of initial monomers were mixed with ethanol in two separate beakers.

### Characterization

The diffuse reflectance infrared Fourier transform (DRIFT) spectra were recorded on the Thermo Nicolet Nexus FT-IR at 4 cm^−1^ resolution, using the special thermal vacuum adapter “Collector II” at 100 °C. The samples were mixed with KBr (1:20).

The nitrogen adsorption/desorption isotherms for all the samples were measured on the “Kelvin-1042” adsorption analyzer (Costech Microanalytical). Before the measurements, the samples were degassed at 110 °C, in the helium atmosphere. The BET specific surface area [13] was evaluated in the 0.03–0.35 range of relative pressures.

The content of amino groups was determined by acid-base titration. Batches of the samples (0.05 g) were treated with a 0.1 M HCl solution (20 mL) for 6 h. The precipitates were removed by a magnet, and supernatant was titrated with 0.1 M NaOH, in the presence of indicator (methyl orange) [14]. Concentration of the amino groups was determined from the difference between the content of protons in solution before and after sorption.

### Cu(II) Adsorption Experiments

The batch mode processing was used to examine adsorption of Cu(II) ions. In every experiment, 0.01 g of adsorbent and 10 mL of copper(II) water solution (in the range of 0.15–15 mmol/L) were contacted for 3 h, at 25 °C, except for the kinetic research, where the contact time was different for each experiment (5–180 min). The ionic strength was maintained by adding 1 M NaNO_3_ solution. Metal concentration in aqueous solution was determined by direct titration of metal ions by EDTA solution (in the range of 0.0125–0.025 M), in ammoniac buffer and murexide as complexometric indicator [15]. The adsorption capacities were calculated according to the following equation:

$$ A=\left({C}_0\hbox{-} {C}_{\mathrm{eq}}\right)\cdot V/m $$

where *A*—adsorption capacity (in mmol/g), *C*_0_ and *C*_eq_—initial and equilibrium concentration of Cu(II) ions (in mmol/L), *V*—total volume of the solution (in L), *m*—mass of the adsorbent (in g).

## Results and Discussion

The magnetite nanoparticles (Fe_3_O_4_) were obtained by co-precipitation of iron(II) and (III) salts in a basic medium, under a nitrogen atmosphere [12]. Five different magnetic amino-functionalized silica-based materials were synthesized and tested as potential adsorbents for copper(II) ions. Each of examined adsorbents was synthesized on the basis of sol-gel procedure, which is detailed described in papers [10, 11]. In order to compare sorption measurements, three different monomers containing different number and structure of amino groups were used to modify surface of magnetic silica materials (Table 1).

The presence of functional groups was confirmed by IR spectroscopy, and their content was calculated using acid-base titration (Table 1). The results obtained by acid-base titrations are very close to those published previously [10, 11]. The small differences in the content of amino group values could be attributed by the nature of sol-gel procedure, in which a number of synthesis factors can influence on the properties of the final material. Therefore, such small deviations may fit in a limit of error. What is more, the obtained materials were examined by nitrogen adsorption/desorption measurements. In the Table 1, values of specific surface area (*S*_sp_) are presented. As before, the values of *S*_sp_ are very similar, when compared with the previous results [10, 11]. Small deviations in the values of *S*_sp_ may be caused by measurement error or small differences in the structure of materials, obtained by sol-gel method.

**Fig. 1:**
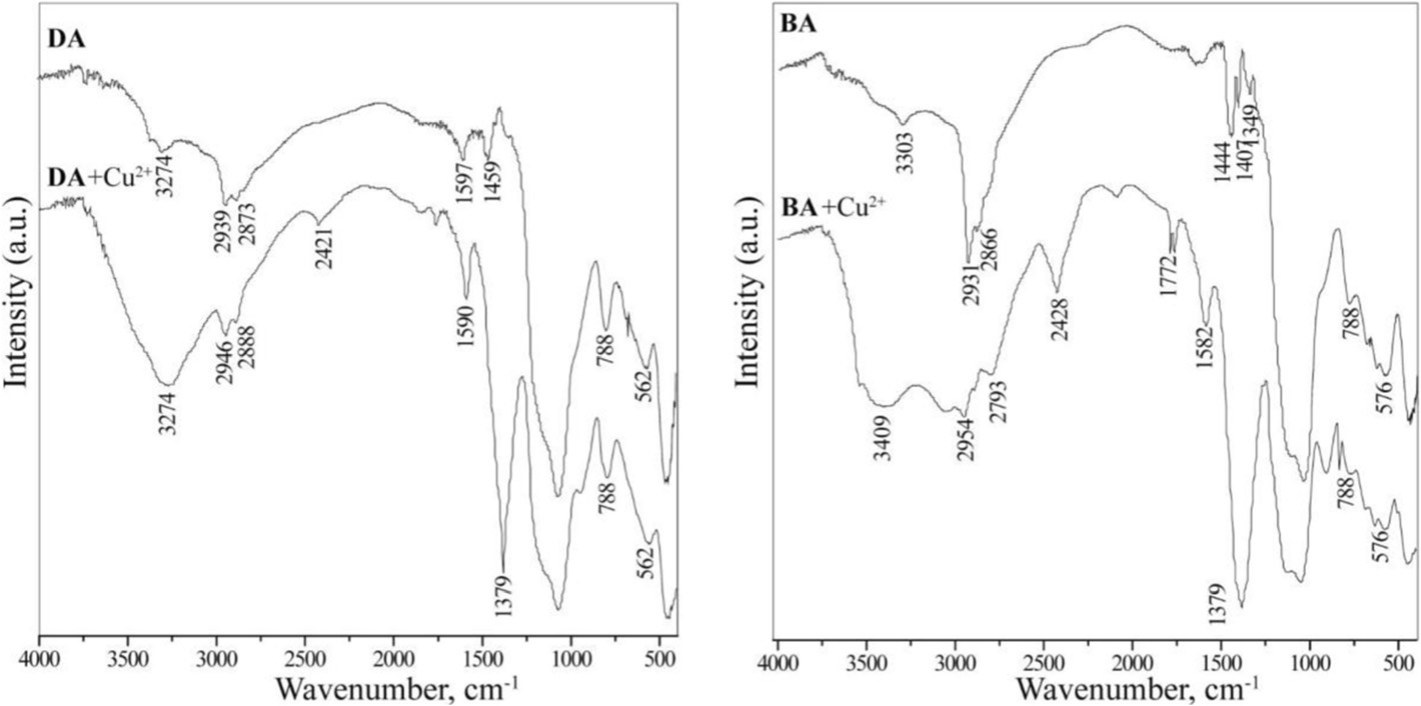
DRIFT spectra of pristine samples and with adsorbed copper(II) ions (100 °C)

DRIFT spectra were recorded by heating the samples to 100 °C, in order to remove adsorbed water, which absorption bands overlap the absorption bands of amino groups (Fig. 1). In all DRIFT spectra, intense and broad absorption band, in the region 550–580 cm^−1^, corresponds to ν(FeO), can be observed. In addition, the DRIFT spectra of the samples with polysiloxane layer contain an intense absorption band with high-frequency shoulder in the region of 1000–1200 cm^−1^, which is characteristic of the ν_as_(SiOSi) stretching vibrations. In the DRIFT spectra of the samples A and DA, the absorption band at 1590–1597 cm^−1^, corresponding to δ(NH_2_) bending of the amino groups, is clearly visible. In addition, at 3270–3370 cm^−1^, two low-intensity absorption bands belonging to ν_s,as_(NH) of amino groups are observed. The presence of propyl chains (Si–CH_2_CH_2_CH_2_–N) in the DRIFT spectra is indicated by a group of absorption bands of weak intensity in the region 1340–1470 cm^−1^: ~1349, ~1407 cm^−1^ (weak), and ~1444-66 cm^−1^, which can be attributed to ɷ(CH_2_), δ(Si–CH_2_), and δ_as_(CH_2_), respectively. Two absorption bands of medium intensity in the region 2850–2940 cm^−1^ are characteristic of symmetric and asymmetric stretching vibrations of C-H bonds. In addition, the DRIFT spectrum of the sample with amino/methyl groups (AM, not presented here) has sharp absorption band at 1273 cm^−1^, which is absent in the DRIFT spectra of other samples and can be attributed to δ_s_(CH_3_) of methyl group bond to a silicon atom [16].

IR results, obtained from IR spectroscopy, clearly show that the surface of the obtained Fe_3_O_4_ particles is coated by polysiloxane layers with amino-containing functional groups.

**Fig. 2:**
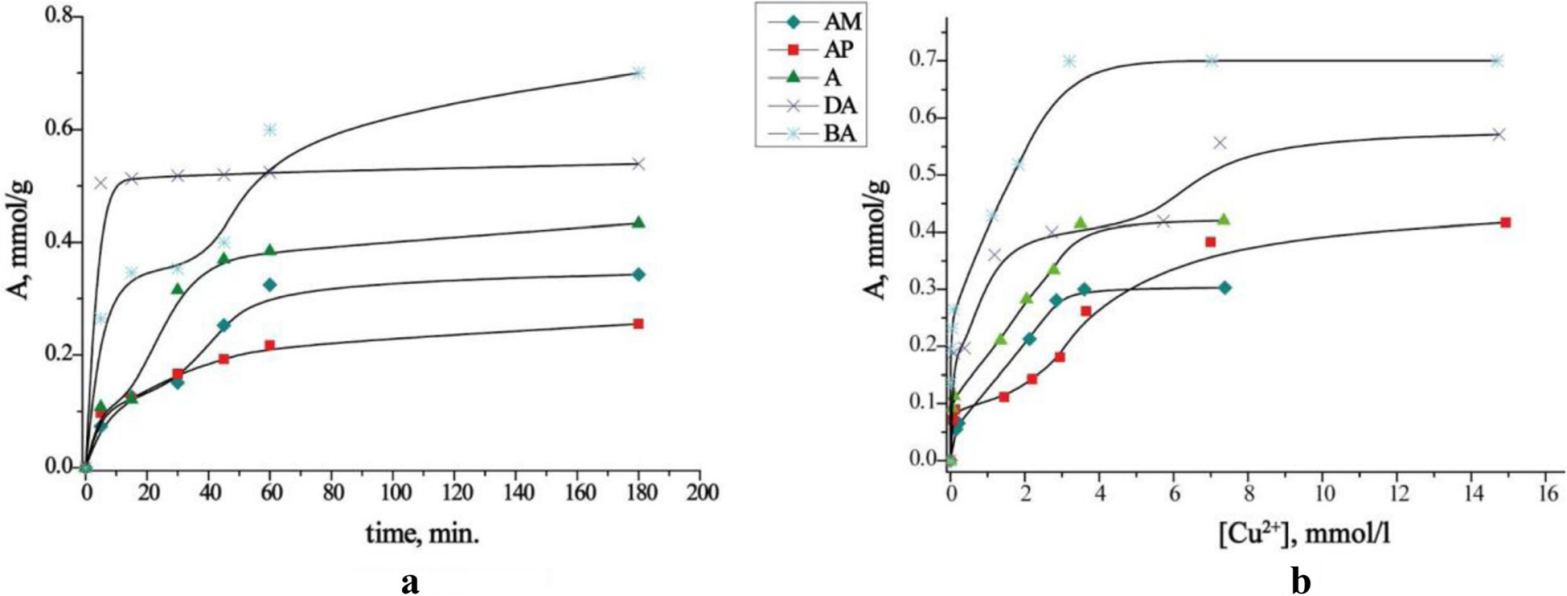
Kinetic curves (**a**) and adsorption isotherms (**b**) of Cu(II) ions on magnetite nanoparticles, functionalized with N-containing groups

**Fig. 3:**
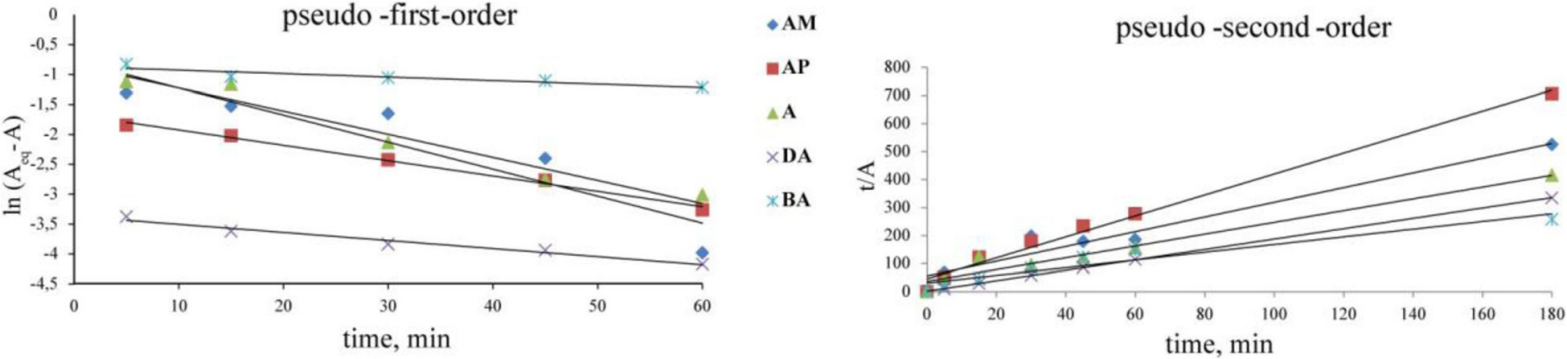
Kinetic curves of Cu(II) ions adsorption in the coordinates of Laherhren’s equation for pseudo-first- and pseudo-second-order processes

Kinetic experiments were performed to estimate the equilibrium time needed to adsorption of Cu(II) ions onto synthesized materials (Fig. 2a). As it is shown in Fig. 2a, adsorption of Cu(II) occurs in two steps for A, AM, BA, and AP samples. The first step involves a rapid metal uptake within the first 30 min of contact. It have to been noticed that the highest value of the *k*_2_ constant and the correlation coefficient is for DA sample. A high content of amino groups ensures almost 98 % of copper(II) ion adsorption from the first minutes. To ensure reaching the equilibrium, the contact time between adsorbent and Cu(II) solutions was set to 3 h. In order to analyze the Cu(II) adsorption kinetics, pseudo-first- and pseudo-second-order equations, as kinetic models [17], were calculated (Fig. 3, Table 2). It can be seen that a_eq_ calculated by the equation of pseudo-second order is close to the experimental one. The high values of correlation coefficients for pseudo-second-order equation show that the removal of Cu(II) ions by functionalized magnetic materials could be attributed to chemisorption.

**Table 2:** Kinetic adsorption parameters obtained by using pseudo-first- and pseudo-second-order models for Cu(II) adsorption

Adsorbent	Pseudo-first order ln (*a*_eq_ − *a*_*t*_) = ln *a*_eq_ − *k*_1_ *t*		Pseudo-second order *t*/*a*_*t*_ = 1/(*k*_2_ ∙ *a*_eq_^2^) + *t*/*a*_eq_		
	*k*_1_	*R* ^2^	*a*_eq_	*k*_2_	*R* ^2^
A	0.039 ± 0.01	0.954	0.381 ± 0.038	0.122 ± 0.039	0.957
AM	0.045 ± 0.001	0.844	0.268 ± 0.009	0.122 ± 0.048	0.949
AP	0.026 ± 0.005	0.993	0.476 ± 0.047	0.307 ± 0.047	0.988
DA	0.014 ± 0.001	0.969	0.54 ± 0.003	1.996 ± 0.599	0.999
BA	0.006 ± 0.001	0.846	0.73 ± 0.114	0.061 ± 0.031	0.915

*a*_*t*_ and *a*_eq_—the amounts of adsorbed Cu(II) ions, at time (*t*) and (*a*_*t*_) equilibrium (in mmol/g); *k*_1_ and *k*_2_—the rate constants of pseudo-first-order (in min^−1^) and pseudo-second-order adsorption process (in g/mmol/min)

The adsorption isotherms and calculated values, using Langmuir [18] and Freundlich [19] isotherm equations, are shown in Fig. 2b and Table 3. While the Langmuir theory assumes that the adsorption is located on identical and energetically homogeneous and equivalent sites, the Freundlich theory treats about heterogeneous nature of adsorbent surface. The high correlation coefficients indicate that the obtained results fitted better to the Langmuir isotherm.

**Table 3:** Parameters of copper(II) adsorption calculated from Langmuir and Freundlich isotherm models

Adsorbent	Langmuir isotherm^a^ *C*_eq_/*a*_eq_ = 1/(*K*_L_ ∙ *a*_m_) + (1/*a*_m_) ∙ *C*_eq_			Freundlich isotherm^b^ lg *a*_eq_ = lg *K*_F_ + (1/*n*) ∙ lg *C*_eq_	
	*a*_m_, mmol/g	*K*_L_, L/mmol	*R* ^2^	*K*_F_	*R* ^2^
A	0.452	1.358	0.958	0.232	0.966
AM	0.345	1.123	0.989	0.142	0.969
AP	0.487	0.336	0.862	0.149	0.846
DA	0.591	1.111	0.981	0.308	0.924
BA	0.712	4.198	0.997	0.453	0.978

^a^ *a*_eq_—the amount of solute adsorbed in the same condition (in mmol/g); *K*_L_—the Langmuir’s constant characterizing the adsorption energy; *C*_eq_—the concentration of solute remaining in solution after equilibrium to be reached (in mmol/L); *a*_m_—the maximum adsorption capacity in the monolayer

^b^ *a*_eq_—the amount of solute adsorbed (in mmol/g); *K*_F_—the Freundlich’s constant, maximum adsorption capacity (in mmol/g); 1/*n*—the Freundlich’s constant characterizing the intensity of adsorption; *C*_eq_—the concentration of solute remaining in solution after equilibrium (in mol/L)

In addition, DRIFT spectra were obtained and analyzed before and after adsorption of copper(II) ions (Fig. 1). As it is seen from the IR spectra, at the 3150–3250 cm^−1^ region, there are visible absorption bands (even when heated sample), characteristic for stretching vibrations of coordinated 3-aminopropyl groups. An intense absorption band at ~1371 cm^−1^ relating to fluctuations anion NO_3_^−^ can be observed [20]. It is worth to noticed that, in the case of samples with adsorbed Cu(II) ions, an absorption band of deformation vibrations of amino groups is shifted toward to the lower frequency region.

It is known that copper(II) ions can form complexes with amino groups as rule 1:2. If in our case we suggest the formation of such complexes, we can calculate, for all samples, that the part of groups is not involved in complexation, but they are still available for protons. It could be connected with the particles specific structure with the surface layers and groups, located on their surface.

**Fig. 4:**
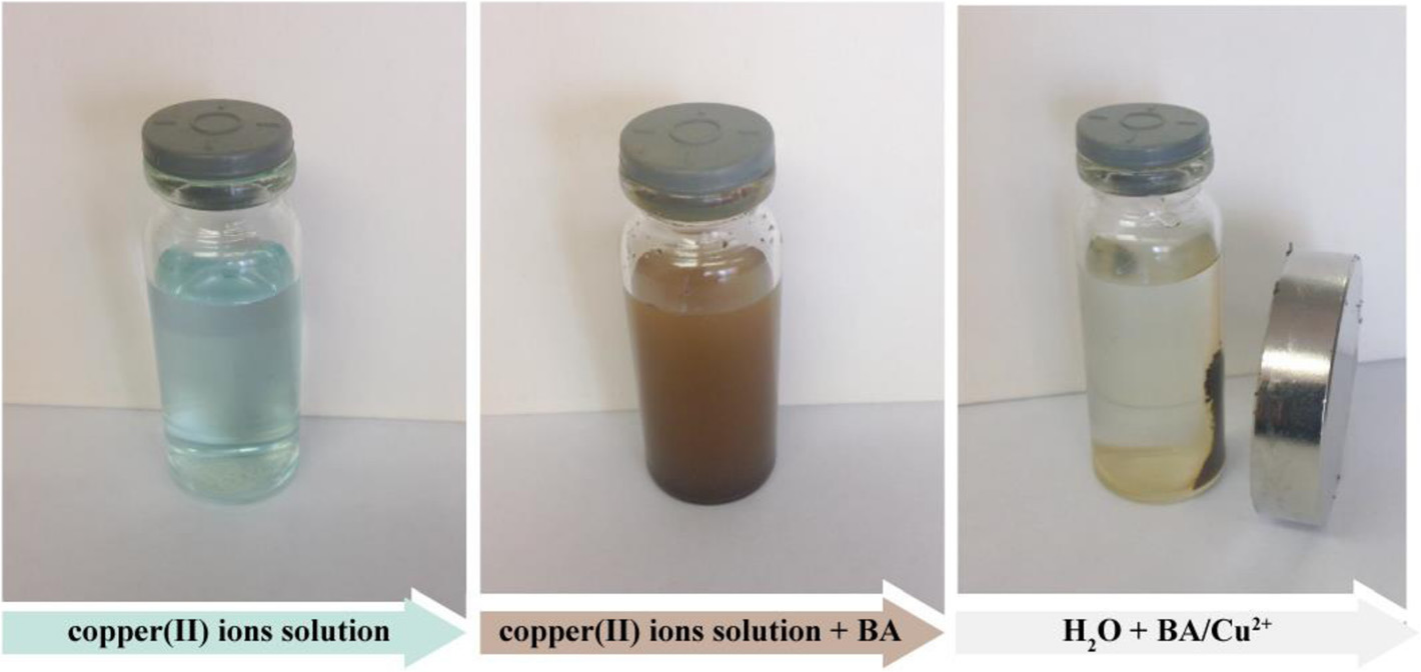
Demonstration of sorption experiment with the sample BA

Figure 4 shows the process of adsorption of copper(II) ions from aqueous solution by BA sample. These photos show adsorption properties of functionalized magnetite sample in relation to copper(II) ions and the preservation of the magnetic properties of the adsorbent even after functionalization process.

In Table 4, comparative characteristics of magnetite composite materials as copper(II) ions adsorbents are summarized. The results presented in this paper indicate that the best adsorption capacity has the sample BA and others materials, synthesized by our research group, when compared to the other references.

**Table 4:** Comparative characteristic of the magnetite/amine-containing composites

Sample	*C*_f.gr._, mmol/g	SSC, mg/g (Cu^2+^)	References
2 N-MSM-e	4.04	32	[3]
Fe_3_O_4_@SiO_2_–NH_2_	1.61	29.8	[4]
Fe_3_O_4_/γ-АПС		4.6	[5]
APTES-NPs	0.69	8.55	[7]
Magnetic chitosan nanoparticle		35.5	[21]
MNP-NH_2_		25.8	[22]
mPMMA		12.8	[23]
A	2.2	26.7	This work
AM	1.6	19.2	This work
AP	1.6	26.5	This work
DA	2.7	36.3	This work
BA	1.9	*44.5*	This work

## Conclusions

Adsorption properties of magneto-sensitive materials in relation to copper(II) ions were studied. It was shown that the amount of adsorbed metal ions depends on the number of functional groups present in the structure of the adsorbent, as well as on their nature. The best adsorption properties showed material functionalized with monomer having secondary amine group (BA), while the fast adsorption kinetic shows sample functionalized with ethylenediamine (DA). Thus, the synthesized materials can be used as efficient adsorbents of Cu(II) ions from aqueous solutions.
